# Defining Landscape Resistance Values in Least-Cost Connectivity Models for the Invasive Grey Squirrel: A Comparison of Approaches Using Expert-Opinion and Habitat Suitability Modelling

**DOI:** 10.1371/journal.pone.0112119

**Published:** 2014-11-07

**Authors:** Claire D. Stevenson-Holt, Kevin Watts, Chloe C. Bellamy, Owen T. Nevin, Andrew D. Ramsey

**Affiliations:** 1 Centre for Wildlife Conservation, University of Cumbria, Ambleside, Cumbria, United Kingdom; 2 Centre for Ecosystems, Society and Biosecurity, Forest Research, Farnham, Surrey, United Kingdom; 3 Centre for Ecosystems, Society and Biosecurity, Forest Research, Roslin, Midlothian, United Kingdom; 4 School of Medical and Applied Sciences, Central Queensland University, Gladstone, Queensland, Australia; 5 School of Biological and Forensic Sciences, University of Derby, Derby, Derbyshire, United Kingdom; University of Queensland, Australia

## Abstract

Least-cost models are widely used to study the functional connectivity of habitat within a varied landscape matrix. A critical step in the process is identifying resistance values for each land cover based upon the facilitating or impeding impact on species movement. Ideally resistance values would be parameterised with empirical data, but due to a shortage of such information, expert-opinion is often used. However, the use of expert-opinion is seen as subjective, human-centric and unreliable. This study derived resistance values from grey squirrel habitat suitability models (HSM) in order to compare the utility and validity of this approach with more traditional, expert-led methods. Models were built and tested with MaxEnt, using squirrel presence records and a categorical land cover map for Cumbria, UK. Predictions on the likelihood of squirrel occurrence within each land cover type were inverted, providing resistance values which were used to parameterise a least-cost model. The resulting habitat networks were measured and compared to those derived from a least-cost model built with previously collated information from experts. The expert-derived and HSM-inferred least-cost networks differ in precision. The HSM-informed networks were smaller and more fragmented because of the higher resistance values attributed to most habitats. These results are discussed in relation to the applicability of both approaches for conservation and management objectives, providing guidance to researchers and practitioners attempting to apply and interpret a least-cost approach to mapping ecological networks.

## Introduction

Effective biodiversity conservation within fragmented landscapes often requires the modelling of connectivity to define the extent of the problem, target conservation activities and to evaluate the impacts of landscape change [1]. Connectivity is defined as the degree to which the landscape facilitates or impedes species movement among resource patches [2]. A landscape consists of a complex, often dynamic, heterogeneous mixture of habitats and land uses which may impact on important ecological processes, such as species movement, habitat selection and survival, and influence behavioural and physiological responses [2]–[5]. The study of the impacts of the matrix on species movement, known as functional connectivity [6], is now the subject of much research within modified and fragmented landscapes [7]. Assessing functional connectivity is commonly used to aid conservation strategies by identifying potential movement pathways across fragmented landscapes for species of conservation concern [8]–[10]. It has also been used to help predict the potential dispersal and movement of invasive species to aid species management by identifying areas to target resources [11], [12].

Geographical Information System (GIS), raster-based least-cost analysis techniques are often used to assess functional connectivity by modelling the impact of permeability of the surrounding landscape matrix on species movement [10]. It has been used in conservation [8]–[10] and invasive species management contexts [11], [12]. For example, the population expansion of the grey squirrel (*Sciurus carolinensis*) in Britain, following its first introduction in 1876 [13], has had negative effects upon the forestry industry and native biodiversity [14]–[16]. In particular, it has occurred simultaneously with the decline and replacement of native red squirrel (*Sciurus vulgaris*) populations through resource competition and disease [14]–[16]. Therefore, an understanding of how grey squirrels utilise and move through the landscape is essential for effective red squirrel conservation and grey squirrel management. By using least-cost modelling it is possible to identify the potential dispersal areas, in addition to the most probable dispersal corridors, to assess the extent of spread [11]. Developing these models involves defining a species' ‘core’ or ‘source’ habitat and assigning resistance values to the surrounding landscape features, based on the actual or perceived impact to species movement at a particular resolution [17]. A cell with a high resistance value is used to represent an area that an individual is unlikely to traverse under typical conditions because of high energy, mortality, or other ecological costs [18]. Using information on a species' maximum dispersal distance, the area around a core habitat patch that is accessible to a species can be mapped with a simple Euclidean buffer. The permeability buffer zone is then taken into account so that the buffer is compressed or stretched according to the cumulative resistance scores assigned to the underlying landscape features. Overlapping buffers therefore signify connections where the species is assumed to be able to move between core habitat patches, forming a functionally connected habitat network.

It is widely acknowledged [4], [18], [19] that a critical step in least-cost modelling is defining resistance values for each type of landscape feature. Beier et al. [19] highlighted three ranked choices for estimating landscape resistance values with the first being the most highly ranked option: 1. empirical animal movement data, genetic distance or rates of inter-patch movements; 2. animal occurrence, density or fitness; 3. literature review and expert opinion. Ideally, resistance values should be informed and parameterised with independent field data, such as extensive mark release recapture studies, actual movement data from radio-telemetry or Global Positioning System (GPS) studies [11], [20], data from experimental studies to record movement through different land cover types [21], or inferred movement data from landscape genetics [9]. However, as these resistance values are species and landscape specific, there is an understandable shortage of such empirical data [22]. Zeller et al. [23] reviewed the different types of data used to parameterize least-cost models and concluded that expert-opinion and occurrence data are most often used. However, they also suggest that comparative studies on the data used to derive resistance values are needed.

Although the use of expert-opinion to parameterise least-cost models is seen as subjective and out performed by values informed by empirical data [24], many studies utilise this type of information to parameterise models [3], [12], [25]. The use of expert-opinion may be appropriate in some cases, such as where there is a particular shortage of empirical data, an urgency to act, or a focus on general principles, focal species or particular species traits. However, in an attempt to make the setting of landscape resistance values less biased and more data-driven, some researchers [26]–[31] are starting to utilise species distribution models, such as MaxEnt [32], to parameterise least-cost connectivity models (defined as option 2 by Beier [19]). This study uses MaxEnt, a species distribution model which utilises maximum entropy principles to predict a species' use of a landscape based upon occurrence data and a selected set of environmental predictors [32]. The habitat suitability indices provided by the models can then be used in calculations [26]–[31] to create least-cost connectivity models. Given that resistance values informed by empirical data are ranked higher [19] and seen to outperform expert-opinion values [24], it is hypothesised that the HSM-informed values will produce a more accurate least-cost network than expert-opinion data. The aim of this study is to investigate how expert-derived resistance values compare against values informed by habitat suitability modelling (HSM). The results of this study provide guidance to researchers and practitioners on the suitability of these approaches for informing management and research objectives relating to both species of conservation concern and invasive species spread.

## Materials and Methods

### Ethical statement

Ethical clearance for this study was approved by the University of Cumbria Ethics Committee, ref 09/17. This was a desk based study with no field work required. Therefore, research permits and licences were not required.

### Study site

**Figure 1:**
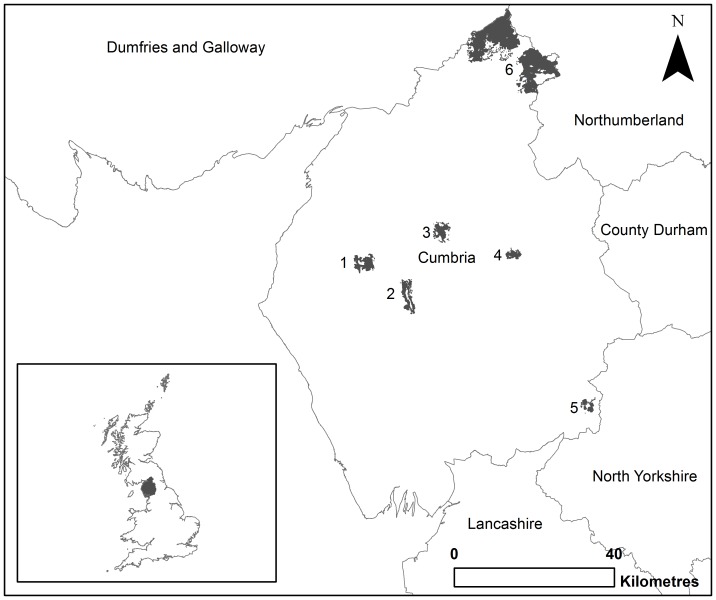
Map of red squirrel reserves in Cumbria and neighbouring counties with reference to its location in the UK. * 1. Whinlatter; 2. Thirlmere; 3. Greystoke; 4. Whinfell; 5. Garsdale/Mallerstang and 6. Kielder (Cumbria proportion of). Boundary lines were obtained through EDINA Digimap Ordnance Survey Service, http://digimap.edina.ac.uk/digimap/home.

To compare expert-derived resistance values against HSM-informed values, grey squirrel within the county of Cumbria UK (Figure 1), are used as the study species. Whilst six large woodlands in Cumbria are designated red squirrel refuge reserves (Figure 1), the grey squirrel remains throughout the county. A number of previous studies have used expert-derived least-cost models to define habitat connectivity for Britain's native red squirrels and invasive grey squirrels [33]–[36], providing expert-opinion on land cover resistance. In addition, Cumbria has an extensive collection of grey squirrel distribution records available with which to create HSM-informed data for comparison. Cumbria covers an area of 6,768 km^2^ and has a sparse population of 490,000 people. The Lake District National Park is located in the centre of Cumbria and has legislation and planning restrictions to conserve the landscape. The National Park Authority are responsible for implementing legislation and planning decisions aimed at conserving the landscape and its species, which means that little has changed regarding land use during the time frame that the species presence data used within this study were recorded (2000–2009). The topography is varied with the Cumbrian Mountain range (≤978 m a.s.l.) that runs approximately west to east across the middle of the county. The majority of land at these higher elevations is used for grazing with little woodland habitat. However, at lower elevations there are numerous woodlands, and other semi-natural habitats, scattered within an agricultural matrix which may provide greater potential for squirrel movement.

### Identifying least-cost networks

Land cover types from a highly accurate and up to date vector land cover map (Ordnance Survey Master Map) were reclassified into 21 broad land cover categories for Cumbria (Table 1). The map was rasterised at 10 m resolution to ensure accurate representation of narrow linear features, such as strips of woodland. All woodland patches were classed as core habitat as squirrels use these areas for nesting and breeding [37], [38]. This map was then parameterised with five alternative expert-derived resistance sets from previous studies (Table 1). The resistance values given in the different studies varied substantially. An additional set of values was developed by the authors by refining Stevenson's [35] scores (referred to as new expert-derived), following a review of the literature and the ecological underpinning of the values that had been applied previously, as described below.

**Table 1:** Land cover resistance values based on previous least-cost modelling studies and resistance set based on expert opinion.

Land cover classification	Stevenson (2008)	Humphrey et al.(2007)	Williams (2008)	Verbeylen et al (2003) *	Gonzales (2000)	New expert-derived	Land cover ranking
Broadleaf	1	0	0	1	1	1	1
Mixed	1	0	0	1	1	1	2
Coniferous	1	0	0	1	1	1	3
Orchard	30	1	4	300	5	16	4
Scrub	1	2	10	10	5	16	18
Coppice	1	1	4	300	5	16	5
Garden	1	1	20	10	5	11	6
Improved/arable/amenity	30	10	20	800	8	40	20
Rough grassland	30	10	20	800	8	40	15
Heath	22	20	20	300	9	37	19
Path	16	50	20	800	1	27	9
Railway verge	12	10	20	800	4	27	11
Road verge	12	10	20	800	4	27	7
Marsh	76	50	50	800	9	91	14
Water	115	50	100	800	999	130	13
Urban	57	50	50	800	1	72	16
Railway	40	50	50	800	9	55	12
Road	12	50	30	800	9	27	8
Track	12	50	30	800	1	27	10
Building	1000	50	50	1000	999	1000	17
Rock	1000	50	50	1000	999	1000	21

*Note the resistance scores given in Verbeylen et al.'s (2003) study are for red squirrels in an urbanised matrix and are used for comparison.

Coniferous, mixed and broadleaved woodland were all assigned the lowest resistance value of 1, as core habitat. Scrub, coppice, orchard, and garden were given relatively low resistance values because they often contain tree species and are commonly used by grey squirrels for commuting [11], [20]. Path, track, road verge, road and railway verge may also be used as commuting corridors, [13], but their use may confer higher mortality risks and therefore they were assigned a relatively high score. Improved/arable/amenity, rough grassland and heath were all attributed higher values still, as squirrel species tend to avoid open habitats [39]. Due to the threat of railways and the difficulty of moving over marsh, water, urban areas, buildings, and rocky areas like cliffs, the high scores assigned in previous studies were maintained.

Least-cost networks were created for each set of resistance values (Table 1) using the least-cost network process outlined in Watts et al. [10]. This network tool analysis utilises ArcView 9.1 and the Spatial Analyst extension (ESRI, Redlands, CA). The first step defined suitable patches of woodland habitat and generated a cost surface raster from the land cover map, by joining the resistance values (Table 1) to the 21 land cover classes. Secondly, the ‘cost-distance’ function in the Spatial Analyst toolbox was used to create a cost-distance surface between woodland patches. The resulting accumulated cost raster was then reclassified to a standardised maximum dispersal distance of 8 km to ensure comparability between the different resistance sets. The ‘region group’ function was used to define each discrete network, using an eight-cell rule so that touching cells, either adjacent and diagonally opposite, within the minimum distance of any given patch were considered part of the same network.

### Deriving resistance scores from habitat suitability modelling

Records of grey squirrel presence were obtained from Save Our Squirrels (http://www.saveoursquirrels.org.uk/). These consisted of 2,281 verified sightings recorded year-round between 2000 and 2009 given by members of the public from both within woodland habitat (35%) and the wider landscape (65%). The grid references and type of habitat the sightings were recorded and verified by Save Our Squirrels. Sightings that were recorded outside of the grey squirrels known distribution range were also verified by contacting the recorder. The points outside of core woodland habitat are believed to relate to landscape use and movement, rather than indicating suitable foraging, breeding or nesting resources [37], [38]. It is these non-woodland records that are used to infer the permeability of the landscape matrix using the habitat suitability modelling software, MaxEnt [40], [32]. MaxEnt assigns each raster cell a Habitat Suitability Index (HSI) based on the environmental conditions at locations where a species has been recorded, using the maximum entropy method [41]. There are three output formats given by the MaxEnt programme: raw, cumulative and logistic; the most easily intuitive logistic HSI scores, which indicate the probability of occupancy ranging between 0–1 and assuming that this is 0.5 at an average site [40], [32], were used in this study.

Both the species records and environmental data were prepared for modelling with MaxEnt. The squirrel data were filtered to remove locations recorded at a resolution of >100 m. Of the remaining 2,008 points, 842 squirrel presences recorded were within the matrix. A categorical land cover raster (gridded data map) was created using the same Ordnance Survey Master Map data and 21 broad land cover categories as previously described (Table 1). However, a coarser resolution of 100 m was used to match the spatial accuracy of the squirrel records. To ensure that linear habitat features were not under-represented, each land cover type was rasterised separately at a 10 m resolution and then aggregated to 100 m using the ‘maximum’ rule. These rasters were mosaicked using ranks that prioritised the classification of each 100 m square containing more than one land cover type (Table 1). All areas of woodland (core habitat), rocks and buildings (highly impermeable) were removed from the land cover map to prevent their incorporation in the model. In an effort to account for sampling bias towards accessible areas, a well-known and common characteristic of species data collected in an *ad-hoc* or non-systematic way [42], all areas over 500 m from a road, track or path were also removed from the map. This left a total of 665 squirrel records that fell within the remaining areas of the land cover map which were used to train and test the habitat suitability model. Each point (located in the south west of the 100 m grid square) was adjusted by 50 m east and 50 m north to locate each point in the centre of the grid square. This was to ensure that the points matched the 100 m raster landscape i.e. were within one cell, not potentially boarding four.

All models were run in MaxEnt Version 3.3.3k, using primarily default settings (regularisation multiplier  = 1; duplicate occurrences removed; maximum number of background points  = 10000, as used in Kramer-Schadt et al. [43]). Five-fold cross validation was used to calculate mean Area Under Roc Curve (AUC) and extrinsic omission rates (the average proportion of test points that fall outside the area predicted to be suitable), following use of the occupancy threshold rule that maximises the sum of test sensitivity and specificity (as recommended by Liu et al., [44]). Residual spatial autocorrelation (rSAC) can inflate measures of model performance [45]–[47] therefore Moran's correlograms were created (1 – predicted HSI for each species record; [48]) using the Spatial Analysis in Macroecology software program (SAM; [49]). Significance of Moran's *I* was calculated using a randomisation test with 9,999 Monte Carlo permutations, correcting for multiple testing.

The response curves, which showed the mean predicted probability of a species' presence (*p*; 0–1 scale) within each land cover type, were used to derive the resistance values for each land cover type. For both the new expert-derived and the HSM-informed values, woodland was given a value of 1, as permeable core habitat, and rock and building given values of 1000, as impermeable land cover types. The remaining land cover type values were inverted and standardised to the same scale as the new expert-derived values, (1–130; using 1-(*p*×130)). These values were then used to identify least-cost networks using the same approach as applied to the new expert-derived resistance scores.

### Comparing resistance scores and resulting habitat networks

An area-minimisation methodology was applied to select for the smallest network that captures the majority (≥90%) of the filtered distribution point data (n = 842). This methodology, derived during this study, was based on the principle that when managing invasive species, areas for control must be defined and defensible to provide successful management [50]. As the grey squirrel population continues to expand in the Cumbria study site, it is important that control efforts are targeted to provide effective management. By identifying habitat networks management can be targeted in these specific areas of the landscape. The larger the habitat networks are the more widespread management would have to be. Therefore, the resistance set which produced networks that include a high proportion of distribution points but a small network area are regarded as the better networks as management can be targeted in these focused areas. In addition a chi square test was used to test whether a significant number of distribution points were within the networks when compared to random points.

The HSM-informed resistance scores and the resulting networks were compared to those created with the new expert-derived set selected by the area-minimisation criteria. A Wilcoxon signed ranks test was used to assess the relative difference between scores. The habitat networks produced were also measured and compared visually and using the distribution points. Distribution points that were within the new expert-derived networks but not within the HSM-informed networks were identified along with the land cover type they were in and vice versa.

## Results

### Habitat Suitability Model performance

The results from five-fold cross-validation test showed that the models performed well (Training sample size  = 532; Test sample size  = 133; Training AUC = 0.80±0.001; Test AUC = 0.78±0.04; Test gain  = 0.70±0.19; Extrinsic omission rate  = 0.23, *P*<0.001), indicating that land cover type provides useful information on the likelihood of grey squirrel presence. No significant residual spatial autocorrelation was found at any distance lag. Moran's I values were <0.05 and statistically insignificant at each distance lag, indicating that the residuals were not spatially autocorrelated.

### Selecting an ‘optimal model’ from expert-derived resistance sets

**Figure 2:**
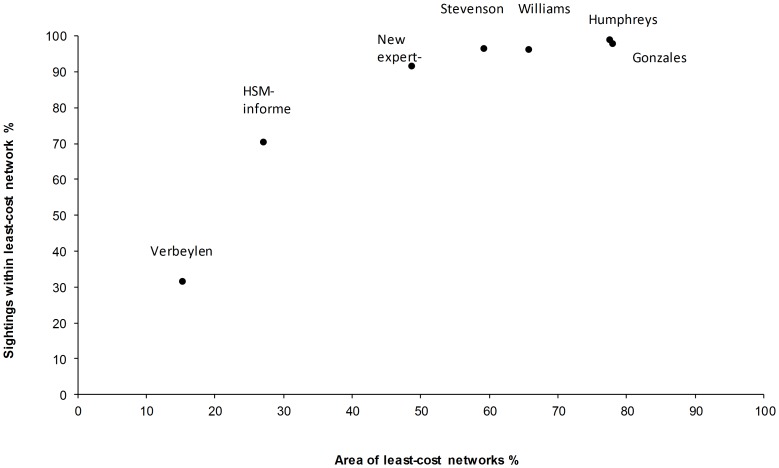
Comparison between expert-derived and Habitat Suitability Model-derived resistance values. Note: values that produce a network with>90% sightings points and the lowest network area is considered the best model for management.

There was considerable variation between the previous studies and new expert-derived resistance sets, with network area ranging from 78% to 15% of the total landscape area, containing between 99% and 32% of the squirrel point data (Figure 2). However, when the networks were tested against the occurrence data within the matrix all resulting networks contained significantly more distribution points than expected by chance (n = 842, Stevenson 2008, χ2 = 623, df. = 1, p<0.001; Humphreys et al. 2007, χ2 = 238, df. = 1, p<0.001; Williams 2008, χ2 = 357, df. = 1, p<0.001; Verbeylen et al. 2003, χ2 = 169, df. = 1, p<0.001; Gonzales 2000, χ2 = 213, df. = 1, p<0.001; new expert-derived, χ2 = 623, df. = 1, p<0.001). Using the area-minimisation methodology, the new expert-derived resistance set was shown to have above 90% of sightings within the networks and the lowest networks area of 49% of the total landscape (Figure 2). This was therefore selected and used for further comparison with the HSM-informed networks.

### Comparing expert-derived and HSM-informed networks

The HSM-informed network had significantly more grey squirrel distribution points within it than expected by chance (n = 842, χ2 = 836, df. = 1, p<0.001). However, the new expert-derived network contained significantly more points than the HSM-informed network (n = 842, χ2 = 185, df. = 1, p<0.001). The majority of land cover types were given higher resistance values using the HSM approach compared to those derived from the new expert-derived values, with relative differences ranging from 7–86% (Table 2). These differences were found to be statistically significant (n = 16, Wilcoxon signed ranks test, *p* = 0.002); water and coppice were the only habitats to be assigned an HSM-informed lower resistance value compared to those derived from the new expert-derived resistance set. The largest differences in the resistance values assigned to habitats by the two approaches were between scrub, tracks, railways and railway verges (Table 2).

**Table 2:** Average probability of grey squirrel presence according to land cover type.

Habitat type	HSM *p* score	HSM-resistance score	New expert-derived resistance score	Difference between HSM and Expert-derived resistance scores
Scrub	0.10	117	16	0.86
Track	0.16	109	27	0.75
Railway	0.17	108	27	0.75
Railway verge	0.17	108	27	0.75
Path	0.34	86	27	0.69
Heath	0.17	108	37	0.66
Garden	0.73	32	11	0.66
Road	0.41	77	27	0.65
Improved/arable/amenity	0.13	113	40	0.65
Rough grassland	0.13	113	40	0.65
Road verge	0.43	74	27	0.64
Orchard	0.77	30	16	0.47
Urban	0.29	92	72	0.22
Marsh	0.17	108	91	0.16
Broadleaf	N/A	1	1	0.00
Coniferous	N/A	1	1	0.00
Mixed	N/A	1	1	0.00
Building	N/A	1000	1000	0.00
Rock	N/A	1000	1000	0.00
Coppice	0.86	15	16	−0.07
Water	0.18	107	130	−0.21

*p* =  mean predicted probability of presence according to habitat type.

**Figure 3:**
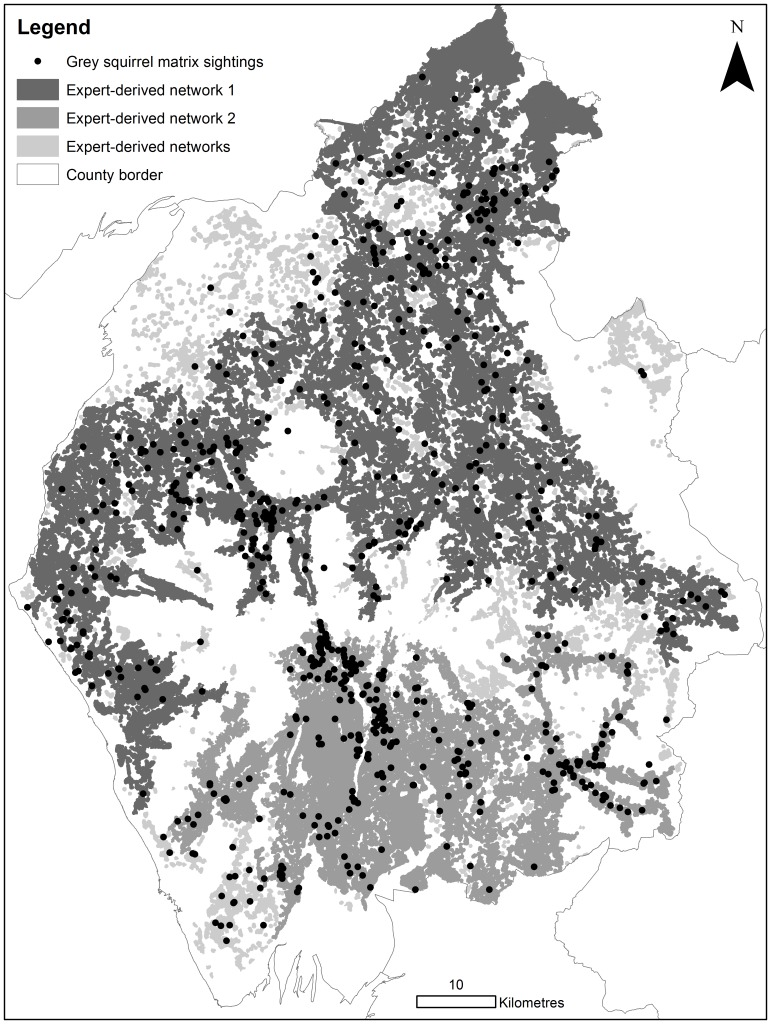
Grey squirrel least-cost habitat networks identified from expert-derived resistance values. Boundary lines were obtained through EDINA Digimap Ordnance Survey Service, http://digimap.edina.ac.uk/digimap/home.

The least-cost model parameterised with the new expert-derived resistance values identified 738 discrete networks, although two of these cover substantial areas; habitat network 1 in the north and habitat network 2 in the south (Figure 3). The mean network size was 4.7 km^2^ (±84.4). These networks accounted for 42% of the Cumbrian land cover (3,518 km^2^) and appear to be separated by the land cover types within the Cumbrian Mountains. The HSM-informed resistance values generated comparatively smaller and more fragmented networks, owing to the higher resistance scores attributed to most habitat types. This network was 55% the size of the new expert-derived network (1,953 km^2^; 34% of land cover) and sat almost entirely inside it, with only 0.2% extending beyond the expert-derived network, over areas of water. The mean network size was 0.3 km^2^ (±5.0) and 5,840 separate networks were identified in Cumbria (Figure 3). Ten of these were relatively large (>20 km^2^). The HSM-informed networks also indicated that networks in the north and south of the county were separated by the Cumbrian Mountains range. Both identified Grizdale Forest and surrounding woodlands as a large, well connected grey squirrel habitat network (Figure 3).

**Figure 4:**
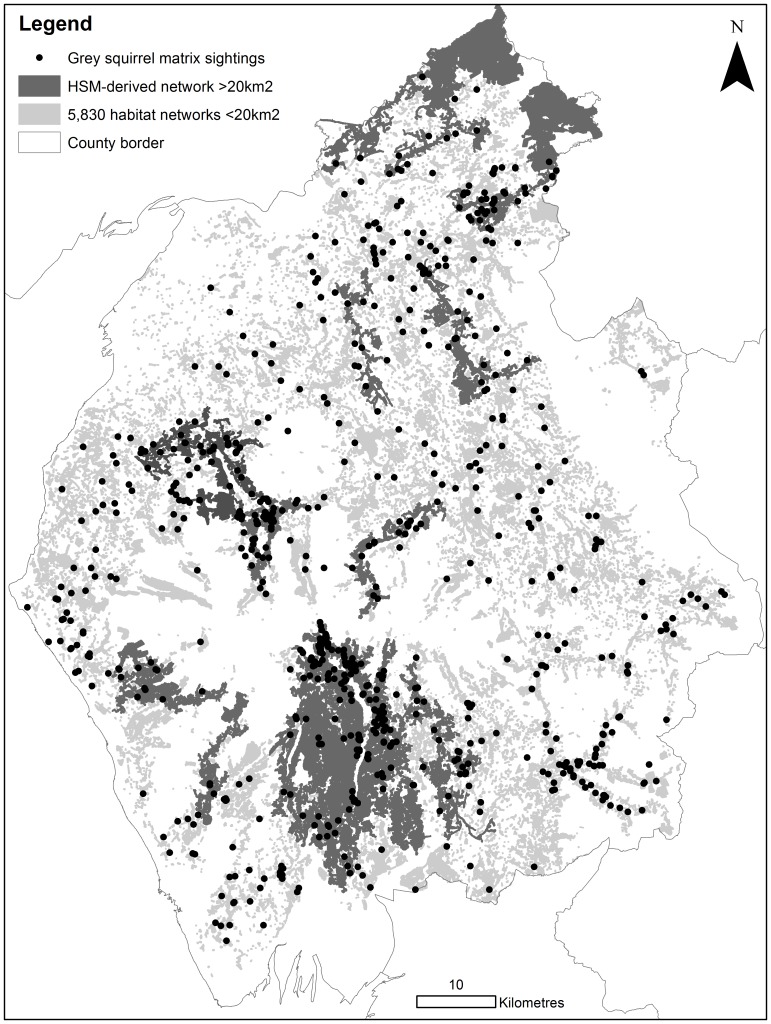
Highly fragmented grey squirrel least-cost habitat networks identified with Habitat Suitability Model-derived resistance values. Boundary lines were obtained through EDINA Digimap Ordnance Survey Service, http://digimap.edina.ac.uk/digimap/home.

The smaller HSM-informed least-cost networks contained 592 (70%) of 842 species records within the habitat network (compared to 772 (92%) using new expert-derived) (Figure 4). As the HSM-informed scores were based upon the actual distribution data it was expected that the resulting networks would include a substantial amount of distribution points. The number of points outside of the HSM-informed least-cost networks was 250; of these points missed by the HSM-informed network 180 were included within the new expert-derived networks. These 180 points were located in improved/arable/amenity land (77%), gardens (8%), rough grassland (6%), urban (3%), road (2%), road verge (1%), tracks (1%), marshland (1%), scrub (1%) or water (1%). The number of points outside of the new expert-derived networks was 70; of these points none were included within the HSM-informed least-cost networks.

## Discussion

When estimating resistance values Beier [19] highlighted three ranked choices. Although using animal movement data, genetic distance or rates of inter-patch movements (option 1) is the preferable option to define resistance values, animal occurrence data (option 2) and/or literature review and expert opinion (option 3) may be the only information available to many researchers and conservationists trying to model functional connectivity in fragmented landscapes. In this study resistance values derived from expert-opinion have been compared to HSM-informed values. Both techniques identified least-cost networks that contained significantly more distribution points than would be expected by chance. However, differences occur between the degree of model assumptions and biases (based on the different types of data), resistance values for certain land cover types and the least-cost networks identified. This has implications for the reliability of using such data in meeting conservation and management objectives.

To derive a set of expert-opinion resistance values it is useful to compare previous resistance values from multiple sources, particularly if the studies have similar species and environmental conditions. The resistance values given in previous studies were highly variable, resulting in varied least-cost habitat network areas and number of distribution points within networks. Although the land cover resistance values given in these studies were for red or grey squirrels, the studies took place in different countries with different regional environmental conditions and large scale and inevitable differences in landscape composition and structure. This may account for the differences in values given and resulting networks. Verbeylen et al [3] in particular was focused on red squirrels and based in an urban area which is very different to the largely-rural and sparsely populated Cumbria. However by assessing the range of different resistance values given in these studies and additional literature on land cover use, the new expert-derived resistance set was created. The area-minimisation method suggests that these values appear to be the best set for management purposes in this area, capturing a high percentage of distribution points within the smallest network area.

The resistance values for the new expert-derived and HSM-informed least-cost models in this study were significantly different from one another. The HSM-informed model provided higher resistance values for most land cover types. The validity of HSM-informed least-cost models may be limited as the probability of occurrence in a particular land cover type does not always equate to the resistance of that land cover type during species movement [19]. In using distribution/occurrence data, certain land cover types may be undervalued when in reality they are used by the species. Conversely there will be land cover types that are overvalued. A key assumption of presence only modelling is that the data has come from random sampling or is representative of the whole landscape [51]. It is questionable whether the degree of bias in presence data can be truly known [51]. Squirrels are well known to use scrub habitat and will use this and linear features to aid dispersal [13], [52]–[54], yet scrub and railway verge (a linear feature) were given high HSM-informed resistance values due to a low number of distribution points. Of the distribution points missed by the HSM-informed networks but included within the new expert-derived networks, 77% were within improved/arable/amenity land cover type. This suggests that the inverted HSM values for this land cover may be too high, and squirrels may be able to cross these hostile areas quickly and undetected. The dispersal distance used for both expert-derived model and the HSM-informed model were set at 8 km. Therefore, it is the higher resistance values given to certain land cover types using the inverted-HSM that led to the identification of smaller and more fragmented networks.

The HSM-informed networks were 45% smaller than the expert-derived networks and were spatially nested inside these networks. The smaller mean size of HSM-informed networks suggests that grey squirrel occurs in a highly fragmented and functionally unconnected landscape. Both models highlight the land cover types of the Cumbrian Mountains as a barrier to movement; the combination of relatively high elevation and intense grazing result in a lack of woodland in the area. Although, some individuals may attempt to cross the barrier, the lack of available habitat will impede dispersal subjecting individuals to high levels of predation and starvation. There are no recorded introductions of the grey squirrel into Cumbria [55], [56] and therefore these animals have been able to spread to their present distribution in the north and south of the county by natural means. The expert-derived model identified two large networks, one in the north and one in the south, suggesting a much more connected landscape.

Studies have suggested that expert-opinion based models perform less accurately than models informed by empirical data [24], [57], [58]. Given that HSM-informed networks are derived from known distribution data, these models could be interpreted as identifying more precise areas in the landscape that are connected for a species. In comparison, the expert-derived networks include those areas where sighting have not been recorded but are judged by experts as permeable to the species during dispersal. Experts may overestimate the importance of certain land cover types erring on the side of caution and therefore rendering the model less accurate [24]. Where actions might require a more precise approach, such as identifying possible protected areas or sites for an efficient and intensive control program, a HSM modelling approach would be appropriate. However, when assessing invasive species it is not just the most likely areas that a species will disperse to, but the entire possible range that needs identified. In an invasive species context, it may be more appropriate to apply a conservative less precise model, such as the expert-derived model, to enable all possible areas of dispersal to be included within the network.

In the case of invasive species the assessment of potential movement and impact is needed as soon as possible to aid management planning. This method is not dependent upon extensive species distribution data and can therefore be produced relatively quickly. Clevenger et al. [24] found that expert only derived resistance values had a weaker correlation with empirical-derived values than literature-derived values. Systematically collecting expert opinion, as promoted by Eycott et al [59], in combination with published data on land cover usage will enable resistance values to be assigned in the initial stages to give an indication of species movements whilst other empirical data is collected where possible. Adriensen et al [18] suggested that once a ‘starter kit’ of resistance values has been identified, sensitivity studies can be initiated and multiple alternative resistance sets can be tested [60]. Once species distribution data is collected, HSM-informed least-cost networks can be identified and used to aid the selection of most likely used sites to focus monitoring or eradication programs. It should not be assumed that using distribution data (option 2 in Beier et al. [19]) to identify resistance values is better or worse than using well developed expert-opinion (option 3 in Beier et al. [19]) as the choice of which method to use may depend upon the aims and objectives of the user and the appropriate precision of the approach.

This paper describes the first step towards developing least-cost habitat networks using *ad hoc* species records and a simple, land cover-based habitat suitability model. It is acknowledged, however, that species respond to their surrounding environment over a range of spatial scales and that both local and landscape features will affect both the suitability of the core habitat and the permeability of the surrounding matrix [5], [61]. More complex models incorporating multiscale information on the terrain, built environment, and the composition, structure and arrangement of habitat patches are likely to provide more accurate and useful models [45], providing predictions at each location, rather than assuming consistent levels of permeability for a particular land cover type. This spatially explicit technique would enable landscape level decision making, improving our ability to identify important networks of habitat and enabling a targeted and informed approach to both conservation and infrastructural development.

### Conclusion

Even though approaches to gather expert opinion are becoming more systematic and robust, it should not be seen as a blanket substitute for empirical data. Empirical data will continue to be important for studies on single species, where there is considerable uncertainty or where there is significant investment in time and money on conservation activities. Conservation planners must be aware of the subjectivity and pitfalls of the different types of data used in least-cost models, without any further validation or sensitivity testing of model values. If expert opinion is the only option available it should be used as a first step by systematically combining multiple expert opinions and published data, but with the knowledge that further assessment of resistance values through sensitivity analysis and empirical data will be needed. Where distribution data is already available, the type of data collection and the subjective translation issues of over and under valuing land cover types must be assessed with expert knowledge or empirical data and explicitly stated in methodologies [51], [62].

This study successfully compared expert-derived and HSM-informed resistance values used in least-cost modelling. Although the results of the models differed, both identified equally useful least-cost networks. For the grey squirrel in Cumbria, both expert-derived and HSM-informed networks have shown that there is a separation between north and south of Cumbria due to the land cover types and lack of habitat of the Cumbrian Mountain range. The expert-derived networks indicate a conservative less precise least-cost network that indicates the potential dispersal range of the grey squirrel and suggests that there may be multiple infiltration routes into the county from the north and south. This conservative expert-derived approach is useful when dealing with invasive or generalist species to identify the potential extend of spread. When assessing endangered or specialist species, or areas that are highly likely to contain target species, the HSM-informed network provides smaller precise networks. These precise networks should be used to inform targeted conservation to increase connectivity for species of conservation concern, or to inform targeted management to prevent the incursion of invasive species. The variable but acceptable precision of both expert-derived and HSM-informed least-cost networks highlights the need to consider data reliability and environmental context when deciding on the most appropriate management of invasive species.

## References

[1] Worboys G, Francis WL, Lockwood M (2009) Connectivity conservation management: A global guide (with particular reference to mountain connectivity conservation). London: Earthscan/James & James.

[2] Taylor PD, Fahrig L, Henein K, Merriam G (1993) Connectivity is a vital element of landscape structure. Oikos 68: 571–573.

[3] Verbeylen G, De Bruyn L, Adriaensen F, Matthysen E (2003) Does matrix resistance influence red squirrel (*Sciurus vulgaris* L. 1978) distribution in an urban landscape? Landscape Ecology 18: 791–805.

[4] Spear SF, Balkenhol N, Fortin MJ, McRae BH, Scribner K (2010) Use of resistance surfaces for landscape genetic studies: Considerations for parameterization and analysis. Mol Ecol 19: 3576–3591. 20723064 10.1111/j.1365-294X.2010.04657.x

[5] Ricketts TH (2001) The matrix matters: Effective isolation in fragmented landscapes. The American Naturalist 158: 87–99. 10.1086/320863 18707317

[6] Tischendorf L, Fahrig L (2000) On the usage and measurement of landscape connectivity. Oikos 90: 7–19.

[7] Crooks KR (2006) Connectivity conservation. Cambridge: Cambridge Univ Pr.

[8] Ferreras P (2001) Landscape structure and asymmetrical inter-patch connectivity in a metapopulation of the endangered Iberian lynx. Biol Conserv 100: 125–136.

[9] Epps CW, Wehausen JD, Bleich VC, Torres SG, Brashares JS (2007) Optimizing dispersal and corridor models using landscape genetics. J Appl Ecol 44: 714–724.

[10] Watts K, Eycott AE, Handley P, Ray D, Humphrey JW, et al (2010) Targeting and evaluating biodiversity conservation action within fragmented landscapes: An approach based on generic focal species and least-cost networks. Landscape Ecology 25: 1305–1318.

[11] Stevenson CD, Ferryman M, Nevin OT, Ramsey AD, Bailey S, et al (2013) Using GPS telemetry to validate least-cost modeling of gray squirrel (*Sciurus carolinensis*) movement within a fragmented landscape. Ecology and Evolution 3: 2350–2361. 23919175 10.1002/ece3.638 PMC3728970

[12] Gonzales EK, Gergel SE (2007) Testing assumptions of cost surface analysis- a tool for invasive species management. Landscape Ecology 22: 1155–1168.

[13] Middleton AD (1930) The ecology of the American grey squirrel (*Sciurus carolinensis* gmelin) in the British isles. J Zool, Lond 100: 809–843.

[14] Gurnell J, Wauters LA, Lurz PWW, Tosi G (2004) Alien species and interspecific competition: Effects of introduced eastern grey squirrels on red squirrel population dynamics. J Anim Ecol 73: 26–35.

[15] Gurnell J, Mayle B (2003) Ecological impacts of the alien grey squirrel (*Sciurus carolinensis*) in Britain. In Bowen CP, editor. MammAliens – A one day conference on the problems caused by non- native British mammals. London: Peoples Trust for Endangered Species/Mammals Trust UK. pp.40–45.

[16] Kenward RE (1983) The causes of damage by red and grey squirrels. Mamm Rev 13: 159–166.

[17] Sawyer SC, Epps CW, Brashares JS (2011) Placing linkages among fragmented habitats: Do least-cost models reflect how animals use landscapes? J Appl Ecol 48: 668–678.

[18] Adriaensen F, Chardon JP, De Blust G, Swinnen E, Villalba S, et al (2003) The application of ‘least-cost’ modelling as a functional landscape model. Landscape and Urban Planning 64: 233–247.

[19] Beier P, Majka DR, Spencer WD (2008) Forks in the road: Choices in procedures for designing wildland linkages. Conserv Biol 22: 836–851. 18544090 10.1111/j.1523-1739.2008.00942.x

[20] Driezen K, Adriaensen F, Rondinini C, Doncaster CP, Matthysen E (2007) Evaluating least-cost model predictions with empirical dispersal data: A case-study using radio tracking data of hedgehogs (*Erinaceus europaeus*). Ecological Modelling 209: 314–322.

[21] Stevens VM, Polus E, Wesselingh RA, Schtickzelle N, Baguette M (2005) Quantifying functional connectivity: Experimental evidence for patch-specific resistance in the natterjack toad (*Bufo calamita*). Landscape Ecol 19: 829–842.

[22] Eycott AE, Stewart GB, Buyung-Ali LM, Bowler DE, Watts K, et al (2012) A meta-analysis on the impact of different matrix structures on species movement rates. Landscape Ecol 27: 1263–1278.

[23] Zeller KA, McGarigal K, Whiteley AR (2012) Estimating landscape resistance to movement: A review. Landscape Ecol 27: 777–797.

[24] Clevenger AP, Wierzchowski J, Chruszcz B, Gunson K (2002) GIS-generated, expert-based models for identifying wildlife habitat linkages and planning mitigation passages. Conserv Biol 16: 503–514.

[25] Chardon JP, Adriaensen F, Matthysen E (2003) Incorporating landscape elements into a connectivity measure: A case study for the speckled wood butterfly (*Pararge aegeris* L.). Landscape Ecology 18: 561–573.

[26] Wang Y, Yang K, Bridgman CL, Lin L (2008) Habitat suitability modelling to correlate gene flow with landscape connectivity. Landscape Ecol 23: 989–1000.

[27] Richards-Zawacki CL (2009) Effects of slope and riparian habitat connectivity on gene flow in an endangered Panamanian frog, *Atelopus varius* . Divers Distrib 15: 796–806.

[28] Decout S, Manel S, Miaud C, Luque S (2010) Connectivity loss in human dominated landscape: Operational tools for the identification of suitable habitat patches and corridors on amphibian's population. Landscape International Conference IUFRO, Portugal.

[29] Wang IJ, Summers K (2010) Genetic structure is correlated with phenotypic divergence rather than geographic isolation in the highly polymorphic strawberry poison-dart frog. Mol Ecol 19: 447–458. 20025652 10.1111/j.1365-294X.2009.04465.x

[30] Decout S, Manel S, Miaud C, Luque S (2012) Integrative approach for landscape-based graph connectivity analysis: A case study with the common frog (*Rana temporaria*) in human-dominated landscapes. Landscape Ecol 27: 267–279.

[31] Howard A, Bernardes S (2012) A maximum entropy and least cost path model of bearded capuchin monkey movement in Northeastern brazil. Intergraph. Available: https://intergraphgovsolutions.com/assets/white-paper/A_Maximum_Entropy-2_Imagery.sflb.pdf Accessed 2013.

[32] Phillips SJ, Anderson RP, Schapire RE (2006) Maximum entropy modeling of species geographic distributions. Ecol Model 190: 231–259.

[33] Gonzales EK (2000) Distinguishing between modes of dispersal by introduced eastern grey squirrels (*Sciurus carolinensis*). MSc Thesis, University of Guelph.

[34] Humphrey J, Smith M, Shepherd N, Handley P (2007) Developing lowland habitat networks in Scotland: Phase 2. Edinburgh: Forestry Commission.

[35] Stevenson CD (2008) Modelling red squirrel population viability under a range of landscape scenarios in fragmented woodland ecosystems on the Solway plain, Cumbria. London: People's Trust for Endangered Species. Available http://insight.cumbria.ac.uk. Accessed 2013.

[36] Williams S (2008) Red squirrel strongholds consultation. Edinburgh: Forestry Commission.

[37] Lowe VPW (1993) The spread of the grey squirrel (*Sciurus carolinensis*) into Cumbria since 1960 and its present distribution. J Zool, Lond 231: 663–667.

[38] Skelcher G (1997) The ecological replacement of red by grey squirrels. In: Gurnell J, Lurz P, editors. The conservation of red squirrels, *Sciurus vulgaris* L. London: People's Trust for Endangered Species. pp.67–78.

[39] Nixon CM, McClain MW, Donohoe RW (1980) Effects of clear-cutting on grey squirrels. Journal of Wildlife Management 44: 403–412.

[40] Phillips SJ, Dudik M (2008) Modeling of species distributions with maxent: New extensions and a comprehensive evaluation. Ecography 31: 161–175.

[41] Phillips SJ, Dudík M, Elith J, Graham CH, Lehmann A, et al (2009) Sample selection bias and presence-only distribution models: Implications for background and pseudo-absence data. Ecol Appl 19: 181–197. 19323182 10.1890/07-2153.1

[42] Warton DI, Renner IW, Ramp D (2013) Model-based control of observer bias for the analysis of presence-only data in ecology. Plos One 8: e79168. 24260167 10.1371/journal.pone.0079168 PMC3832482

[43] Schadt S, Knauer F, Kaczensky P, Revilla E, Wiegand T, et al (2002) Rule-based assessment of suitable habitat and patch connectivity for the Eurasian lynx. Ecol Appl 12: 1469–1483.

[44] Liu C, White M, Newell G (2013) Selecting thresholds for the prediction of species occurrence with presence-only data. J Biogeogr 40: 778–789.

[45] Bellamy C, Scott C, Altringham J (2013) Multiscale, presence-only habitat suitability models: Fine-resolution maps for eight bat species. J Appl Ecol 50: 892–901.

[46] Merckx B, Steyaert M, Vanreusel A, Vincx M, Vanaverbeke J (2011) Null models reveal preferential sampling, spatial autocorrelation and overfitting in habitat suitability modelling. Ecol Model 222: 588–597.

[47] Veloz SD (2009) Spatially autocorrelated sampling falsely inflates measures of accuracy for presence-only niche models. J Biogeogr 36: 2290–2299.

[48] De Marco P, Diniz-Filho JA, Bini LM (2008) Spatial analysis improves species distribution modelling during range expansion. Biol Lett 4: 577–580. 18664417 10.1098/rsbl.2008.0210 PMC2610070

[49] Rangel TF, Diniz-Filho JAF, Bini LM (2010) SAM: A comprehensive application for spatial analysis in macroecology. Ecography 33: 46–50.

[50] Zalewski A, Piertney SB, Zalewska H, Lambin X (2009) Landscape barriers reduce gene flow in an invasive carnivore: Geographical and local genetic structure of American mink in Scotland. Mol Ecol 18: 1601–1615. 19302354 10.1111/j.1365-294X.2009.04131.x

[51] Yackulic CB, Chandler R, Zipkin EF, Royle JA, Nichols JD, et al (2013) Presence-only modelling using MaxEnt: When can we trust the inferences? Methods in Ecology and Evolution 4: 236–243.

[52] Taylor KD, Shorten M, Lloyd HG, Courtier FA (1971) Movements of the grey squirrel as revealed by trapping. J Appl Ecol 8: 123–146.

[53] Fitzgibbon CD (1993) The distribution of grey squirrel dreys in farm woodland: The influence of wood area, isolation and management. J Appl Ecol 30: 736–742.

[54] Wauters LA, Gurnell J, Currado I, Mazzoglio P (1997) Grey squirrel *Sciurus carolinensis* management in Italy - squirrel distribution in a highly fragmented landscape. Wildlife Biology 3: 117–123.

[55] Shorten M (1954) Squirrels. London: Collins.

[56] Shorten M (1957) Squirrels in England, Wales and Scotland, 1955. J Animal Ecol 26: 287–294.

[57] Pearce J, Cherry K, Whish G (2001) Incorporating expert opinion and fine-scale vegetation mapping into statistical models of faunal distribution. J Appl Ecol 38: 412–424.

[58] Seoane J, Bustamante J, Diaz-Delgado R (2005) Effect of expert opinion on the predictive ability of environmental models of bird distribution. Conserv Biol 19: 512–522.

[59] Eycott AE, Marzano M, Watts K (2011) Filling evidence gaps with expert opinion: The use of Delphi analysis in least-cost modelling of functional connectivity. Landscape Urban Plann 103: 400–409.

[60] Rayfield B, Fortin MJ, Fall A (2010) The sensitivity of least-cost habitat graphs to relative cost surface values. Landscape Ecol 25: 519–532.

[61] Wiens JA (1989) Spatial scaling in ecology. Funct Ecol 3: 385–397.

[62] Beier P, Majka DR, Newell SL (2009) Uncertainty analysis of least-cost modeling for designing wildlife linkages. Ecological Applications 19: 2067–2077. 20014579 10.1890/08-1898.1

